# Selection of a Real-Time PCR Housekeeping Gene Panel in Human Endothelial Colony Forming Cells for Cellular Senescence Studies

**DOI:** 10.3389/fmed.2019.00033

**Published:** 2019-03-11

**Authors:** Kiran J. McLoughlin, Edoardo Pedrini, Meabh MacMahon, Jasenka Guduric-Fuchs, Reinhold J. Medina

**Affiliations:** Centre for Experimental Medicine, School of Medicine, Dentistry, and Biomedical Science, Queen's University Belfast, Belfast, United Kingdom

**Keywords:** housekeeping gene, ECFCs, senescence, RT-qPCR, computational biology

## Abstract

Endothelial Colony Forming Cells (ECFCs) represent a subset of endothelial progenitors with well-documented vasoreparative capacity. However, cellular senescence, which occurs due to aging, diabetes, smoking, or tissue inflammation, renders these cells dysfunctional. Therefore, there is growing interest in studying expression of senescence markers in ECFCs. RT-qPCR is the most commonly used technique to quantify gene expression and the proper choice of reference genes used for data normalization is critical for accurate quantification. It has been reported that the expression of commonly used housekeeping genes is often unstable in senescence. To identify the most suitable reference genes for ECFC senescence studies, we analyzed a microarray dataset, which compared the gene expression between proliferating and senescent ECFCs. In addition to replicative senescence, the data included X-ray-induced and Etoposide-induced senescence. We used the geNorm algorithm to identify the most stable genes across all studied conditions. Gene Ontology analysis found that the most stable genes belonged to the KEGG category of Genetic Information Processing. The optimal combination of housekeeping genes for ECFC senescence was found to include four ribosomal protein genes; RPL13, RPL31, RPL37, and RPL30. The RT-qPCR validation confirmed that normalization with our novel panel was more sensitive in identifying senescence markers compared to commonly used genes such as ACTB, UBC, and GAPDH.

## 1. Introduction

Endothelial progenitor cells (EPCs) are defined as cells circulating in blood with the capacity to differentiate into endothelial cells and contribute to new blood vessel formation. There has been controversy and confusion in the literature when studying EPCs. We and others have proposed nomenclature guidelines for precise terminology (1). A subtype of EPC which is clearly defined by their immunophenotype and functional properties are endothelial colony forming cells (ECFCs). ECFCs exhibit both progenitor and endothelial characteristics and have been considered the *bona fide* EPC (2). There is agreement that ECFCs have remarkable vasoreparative potential and consequently represent an ideal candidate for cell therapy (3–5). ECFCs are isolated as small clusters of cells and therefore, require *in vitro* cell number amplification from tens to millions to meet numbers needed for a cell therapy. Our data demonstrated that this is feasible, and we can expand ECFC numbers from 100,000 to 2.5 billion cells in 14 days. Although ECFCs have significant proliferative potential, they have a Hayflick limit and undergo replicative senescence (6). In order to characterize the senescence program in ECFCs at the gene expression level, there is a requirement to optimize and validate reference genes. While microarray and RNA-seq are high-throughput technologies that allow genome-wide assessment of transcriptomes, reverse transcriptase real time quantitative polymerase chain reaction (RT-qPCR) remains the most frequently used methodology for small scale studies of gene expression. RT-qPCR is extremely sensitive, has a broad dynamic range, is fast and highly reproducible (7). Despite these advantages, RT-qPCR accuracy is highly dependent on the choice of reference genes (8). These internal controls, also known as housekeeping genes, are used as the normalization factor and therefore its expression ideally should not be affected by experimental treatments. Poor choice of housekeeping genes has a major impact on results and could lead to generation of misleading information. Minimum Information for Publication of Quantitative Real-Time PCR Experiments (MIQE) guidelines have indicated the need for validation of the housekeeping gene choice to ensure stable expression across the experimental settings (9). The general assumption that “classical” housekeeping genes are appropriate is not justified because it has been demonstrated that the expression GAPDH and β-actin change under certain *in vitro* experimental conditions, as well as with respect to source material. For example, Glare and colleagues found that β-actin and GAPDH expression is reduced in both broncoalveolar lavage fluid cells and endobronchial biopsy tissue in asthmatics compared to healthy controls (10, 11). In particular, several studies have highlighted that the expression of common housekeeping genes is often unstable in aging and cellular senescence (12–14). Therefore, considering that aging is associated with major metabolic and structural changes in cell phenotypes, it is critical to identify the most stable gene normalizers for senescence studies. Here, we established and validated a panel of housekeeping genes for cellular senescence studies using ECFCs.

## 2. Materials and Methods

### 2.1. ECFC Isolation and Characterization

ECFCs were obtained from human umbilical cord blood with appropriate maternal consent and under ethical approval in accordance with the Declaration of Helsinki. The mononuclear cell fraction was isolated by density gradient fractionation. Umbilical cord blood was diluted using Alsever's solution (Sigma-Aldrich) and carefully layered on Histopaque-1077 (Sigma-Aldrich) in a 1:1 ratio. The fraction at the interphase between the Histopaque and the plasma was carefully removed using a transfer pipette and resuspended in EGM-2 (Lonza Ltd.) supplemented with 20% fetal bovine serum (Hyclone), and plated in 24-well NUNC tissue culture plates precoated with rat tail collagen type I (BD Biosciences). ECFCs were characterized by immunophenotyping for CD31, CD105, CD14, and CD45 (eBioscience) using an Acoustic Focusing Cytometer (Attune NxT, Life Technologies) following already established methodology (5). For all experiments, ECFCs at passage 9 were used.

### 2.2. Induction of Senescence

Both replicative and stress-induced senescent ECFCs were generated for this study. Stress-induced senescence was induced by treating ECFCs with 1 μM Etoposide (Sigma) for 4 days. Media was replaced with fresh EGM-2 supplemented with 10% fetal calf serum (Gibco) and ECFCs were cultured for an additional 4 days to establish senescence, before RNA was extracted using Maxwell RSC automated RNA extraction system (Promega). For X-ray induced senescence cells were treated with 10 Gy and cultured for 5 days to allow establishment of senescence. For replicative-induced senescence, cells were allowed to grow until their Hayflick limit was reached. Quiescence was induced via contact inhibition by allowing cells to reach and maintain 100% confluency for 5 days before RNA was extracted using Maxwell RSC automated RNA extraction system (Promega).

### 2.3. Microarray

Total RNA was extracted using miRNeasy Mini RNA extraction kit (Qiagen). Total RNA from each sample was quantified by the NanoDrop ND-1000 and RNA integrity was assessed by Agilent 2100 Bioanalyser. The microarray hybridization was performed by Arraystar (Rockwille, US) following established protocols. Microarray data was deposited in GEO with accession number GSE125792.

### 2.4. Quantitative Real Time PCR

RNA was reverse transcribed with the High-Capacity cDNA Reverse Transcription kit (ThermoFisher). Primer sequences shown in Table S1 were designed using Primer BLAST and experimentally validated; only primers matching the MIQE guidelines (Table S2) were used (9). RT-qPCR was performed on Roche LightCycler 480 with the Power SYBR-green PCR Master Mix (ThermoFisher), following the manufacturer instructions. Amplification reactions were done in three technical replicates. Following a polymerase activation step at 95°C for 15 min, 40 cycles, including denaturation at 94°C for 10 s; annealing at 58°C for 10 s, and amplification at 72°C for 10 s were performed. Fluorescent signals generated during PCR amplification were monitored and analyzed with LightCycler 480 software (Roche). The expression level was estimated using 35 cycles as limit of detection (LOD). For both microarray data and RT-qPCR data, 3 biological replicates were used; however, different biological replicates were used between techniques (i.e., microarray used 3 different biological replicates to those used for RT-qPCR). Therefore, independent samples were used for RT-qPCR validation because this provides higher confidence to generalize results as 6 different biological replicates tested using two techniques yielded similar results.

### 2.5. GeNorm Implementation

Stability of gene expression was assessed using the selectHK() function form the NormqPCR package (15). The source code applied is presented below:


  # library(BiocInstaller)
  # biocLite("NormqPCR")
  library(NormqPCR)
  input_file  < - "input.csv"
  df_test  < - read.csv(paste0("~/folder/",
  input_file))
  
  # identify the numeric varible
  # df_test < -df_test[c(1:100,nrow(df_test)),]
  id_test  < - unlist(lapply(df_test,is.numeric))
  mat2  < - as.matrix(df_test[-nrow(df_test),
  id_test])
  str(mat2)
  raw2  < - new("qPCRBatch", exprs = mat2,
   featureCategory =
              as.data.frame(array("OK",
               dim=dim(mat2))))
  sampleNames(raw2)  < - colnames(df_test)[-1]
  featureNames(raw2)  < - as.character
  (df_test[,1][-nrow(df_test)])
  head(exprs(raw2))
  t  < - Sys.time()
  out  < - selectHKs(raw2, method = "geNorm",
                 Symbols = featureNames(raw2),
                  minNrHK = 2, log = T)
  d < -Sys.time()-t
  # print the time enlapsed
  d
  saveRDS(object = out,file = paste0("~/out_",
  input_file,".rds"))


### 2.6. Statistical Analysis

Data handling and statistical analysis was done using R (16). Unless otherwise specified, the alpha value for statistically significant was set to 5%. The tests were performed using the default parameters and in a two-sided manner. Where appropriate, *p*-values were corrected for multiple testing. To calculate the confidence interval of the correlation coefficient, the cor.test() function from the “stats” package (16) has been used.

## 3. Results

### 3.1. Characterizing Gene Stability From the ECFC Transcriptome in a Cellular Senescence Investigation Using geNorm

**Figure 1:**
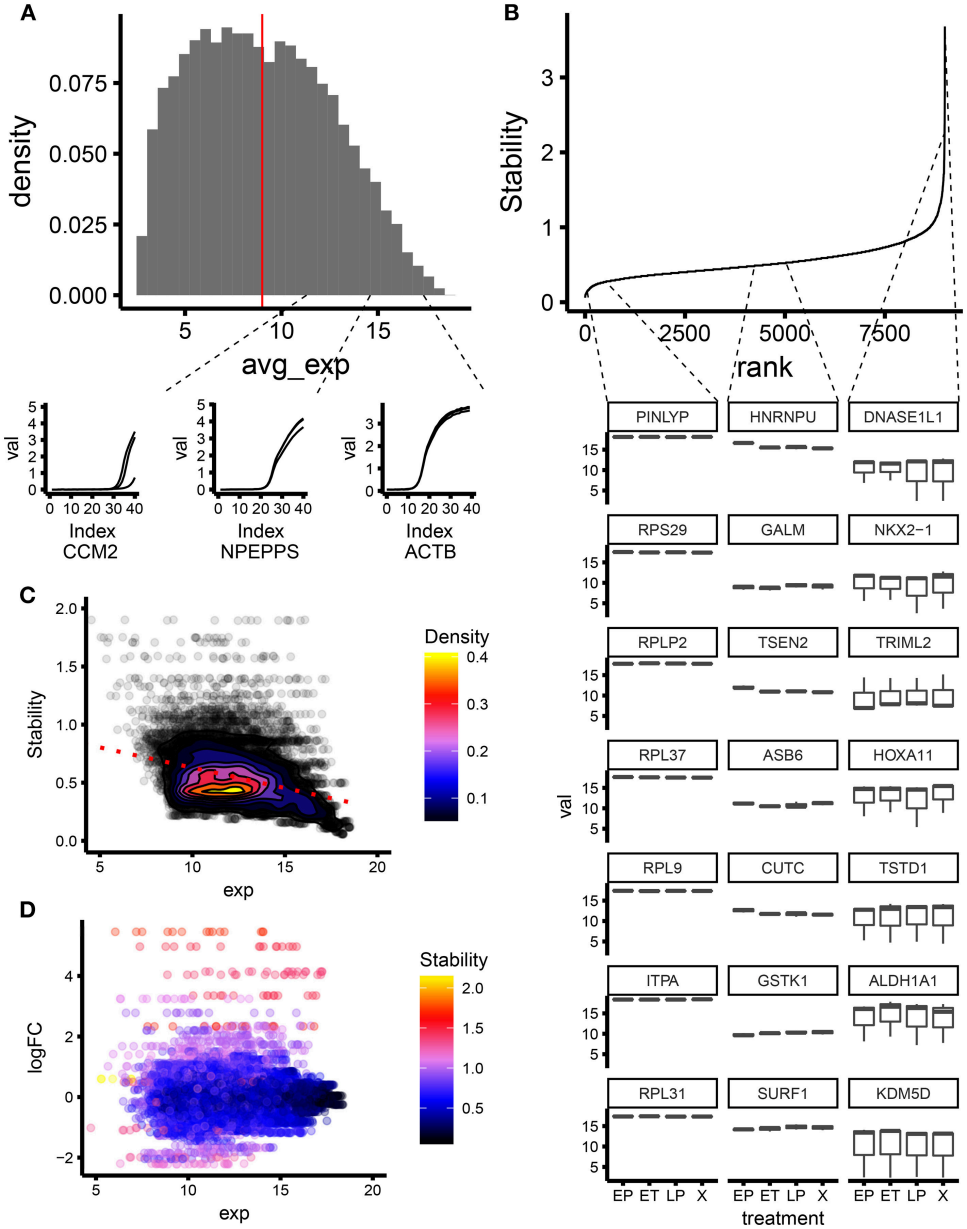
Screening for stable genes using geNorm on microarray data for an investigation of ECFC senescence. **(A)** Distribution of normalized gene expression from microarray data. The red line defines the arbitrary threshold used to filter the dataset. In the lower panel, the qPCR amplification plots for some genes along the distribution are shown. Genes with expression level close to the threshold are close to the LOD of our experimental setup. **(B)** Stability vs. ranking obtained from geNorm. In the lower panel, the distributions of expression levels (from the microarray) for some genes along the ranking are shown. Genes with lower rank have higher within and between treatment variability in gene expression. **(C)** Scatter plot of stability vs. expression (from microarray). The dotted line shows the trend in the dataset; genes with a higher expression level seem to have a lower stability score i.e., more stable. **(D)** MA plot showing level of expression vs. expression fold change. GeNorm stability score depicted using a colored scale.

To identify ECFC genes with the most stable expression during cellular senescence, we analyzed a microarray dataset of non-senescent and senescent ECFCs. Senescence programs investigated were replicative, X-ray induced, and Etoposide-induced. Since microarray and RT-qPCR are two different techniques in terms of dynamic range and limit of detection (17), and to avoid the selection of candidate housekeeping genes that were difficult to detect within our RT-qPCR experimental setup, we decided to filter the dataset focusing on genes with high expression. After normalization, the microarray dataset provided values for average expression of 18,667 probes (Figure 1A). We screened for the level of expression by RT-qPCR along the overall distribution defined by the microarray. Based on these results (Figure 1A bottom panel), we decided to use the top 9,000 probes for further analysis. This threshold was applied as it was both a combination of the halfway point of the microarray data and the point at which transcripts could be unequivocally assessed *in vitro* using RT-qPCR. We applied the geNorm algorithm using the normalized expression level of the probes in the microarray. GeNorm is an algorithm developed by Vandesompele and colleagues which can be used to assess the level of variation in gene expression between different experimental conditions. It works by taking the standard deviation of the genes between experimental conditions and comparing it to the other genes within the dataset. The least stable gene is then excluded and the process is repeated, until two genes remain at the end. Full details can be found in Vandesompele et al. (18). It is important to highlight that the geNorm algorithm provides a stability score, which is inversely proportional to the common idea of stability; therefore, a lower value depicts higher stability. GeNorm indicated that 8616 probes had stability values lower than 1; and 4,444 transcripts had stability values lower than 0.5 (Figure 1B). Furthermore, we confirmed results by visualizing the expression of some genes at different stability ranking levels. As expected, by moving down the ranking, both inter- and intra- variability of the gene expression increased (Figure 1B bottom panel). Interestingly, we noticed a general trend in the dataset; if a gene is more highly expressed, then it seems more likely to be ranked as stable as shown by the dotted red line (Figure 1C). This is also highlighted by the MA plot showing that genes with the higher expression (above 15 variance stabilizing transformation VST units) are the most stable (Figure 1D). When choosing and designing a panel of housekeeping genes, the most stable transcripts from different biologically relevant categories (defined by GO term) were identified from microarray analysis using the geNorm algorithm and validated with PCR. Transcripts with middle and low stability were also sampled at random in order to establish variances in expression based on differing stability values. In summary, we applied the well-established geNorm algorithm to a subset of ECFC senescence microarray dataset to screen for stable targets.

### 3.2. Most Stable Genes Belong to Genetic Information Processing

**Figure 2:**
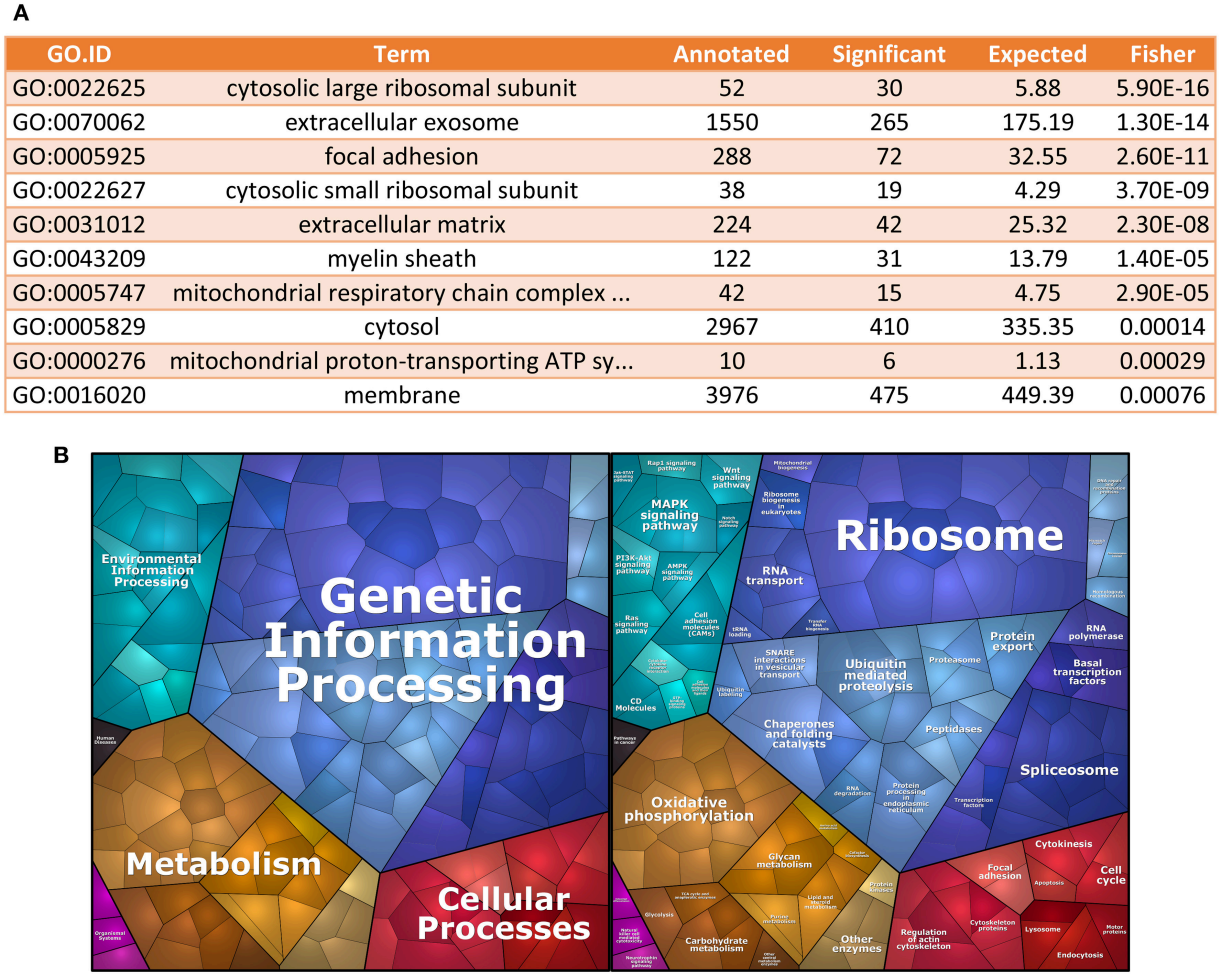
Gene Ontology analysis for most stable genes. **(A)** GO enrichment analysis. The table shows the top 10 GO terms for the Cellular Component domain, using the top 1,000 most stable genes in the dataset. Ribosomal proteins seem to be the most enriched component within the most stable genes. **(B)** Proteomap of the most stable genes. The terms shown in the Voroni plot are based on the KEGG Pathways gene classification. Each gene is shown by a polygon and functionally related proteins are arranged in common regions.

Starting from the geNorm-based results, we used the top 1000 genes, ranked by stability among all senescence samples, to perform GO analysis (19, 20), using Cellular Component domain. We found that GOs for cytosolic large and small ribosomal subunits were among the most enriched cellular components in terms of stability (Figure 2A). To further characterize the identity of the most stable genes, we also performed enrichment analysis using KEGGs terms (21, 22). We found that mRNAs coding for proteins involved in genetic information processing, which include ribosomal protein genes, were among the most stable ECFC genes in the dataset used (Figure 2B).

### 3.3. Four Ribosomal Protein Genes RPL13, RPL31, RPL37, and RPL30 Provide the Optimal Combination of Housekeeping Genes to Normalize Gene Expression in ECFC Senescence Studies

**Figure 3:**
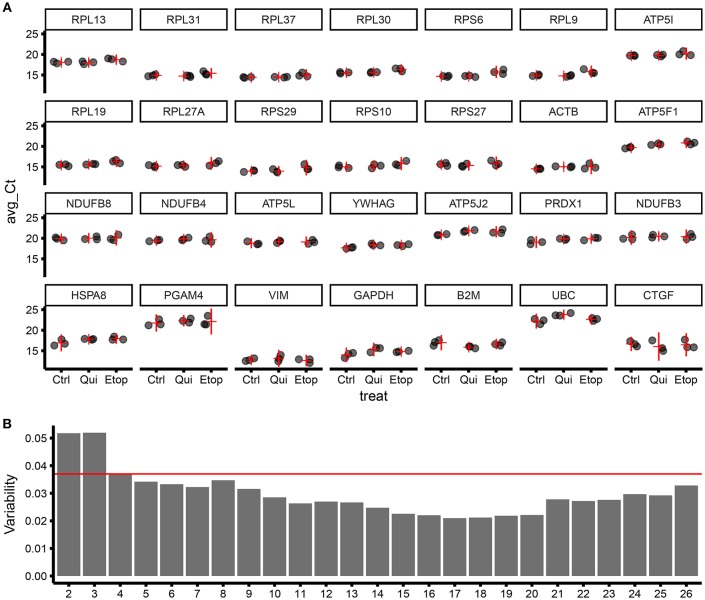
Validation of gene stability from geNorm results using RT-qPCR for 28 candidate housekeeping genes.**(A)** Level of expression (qPCR) for different genes ordered by stability (starting from the most stable; by row; top to bottom), expressed as Ct of three biological replicates (for each biological replicate, three technical replicates were produced). The horizontal red line is the estimate of the average expression for the studied gene in the specific treatment group. The vertical red line is the 95% confidence interval of the estimate of the average expression. The variability within and between treatment increase moving from top-left to bottom-right. Ctrl, control ECFCs; Qui, quiescent ECFCs; Etop, Etoposide-induced senescent ECFCs. **(B)** Bar plot showing sample variability by the number of genes used for calculating the normalization factor (NF).

To refine our ECFC housekeeping gene selection and establish the ideal number of housekeeping genes to be used in a panel, we designed and successfully validated RT-qPCR primers for 28 target genes (Table S1), including targets from geNorm screening of microarray data plus “classical” housekeeping genes i.e., ACTB, GAPDH, and UBC. Those were further tested using different RNA samples including non-senescent, quiescent, and senescent ECFCs. RT-qPCR results were analyzed with geNorm and ranked according to their stability (Figure 3A). As expected, this independent experiment confirmed ribosomal genes as the most stable and validated RPL13, RPL31, RPL37, RPL30, and RPS6 as the top 5 most stable genes among the 28 candidates. In addition, since MIQE (9) guidelines recommend to use at least two housekeeping genes for accurate normalization of gene expression in qPCR experiments; we used the geNorm algorithm to calculate the variability provided by the normalization factor using different number of housekeeping genes. Interestingly using our data and housekeeping candidates, we found that, as expected, the higher the number of genes used, the lower the variability; but from our 28 candidates, we reached a maximum low variability with 17 genes; as using more showed increased variability. In addition, the pairwise variation using the top two housekeeping genes was already below the suggested threshold of 0.15. Nevertheless, results from geNorm variability highlighted that there was an evident improvement in the pairwise variation using 4 genes in the definition of a normalization factor; however, there was a minimal improvement when using 5 instead of 4 genes (Figure 3B). This is also supported by comparing the correlation of the NFs using 3 vs. 4 genes and 4 vs. 5 genes Figure S1).

### 3.4. The Normalization Factor From Our Housekeeping Panel Is Different to the Normalization Factor Using Classical Housekeeping Genes

**Figure 4:**
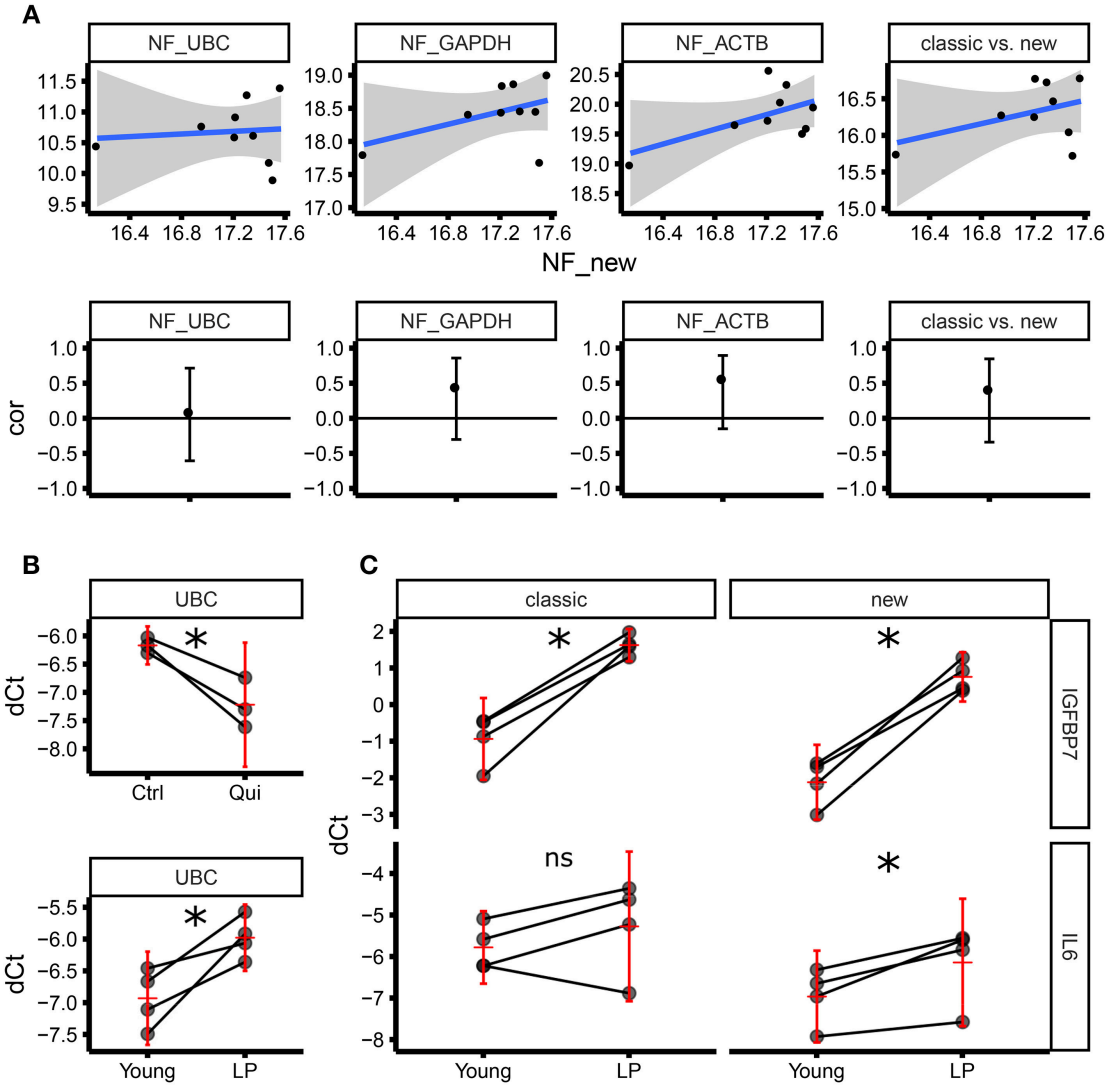
Normalization factor using “classic” housekeeping genes does not correlate with our new geNorm-based housekeeping panel.**(A)** The upper panel shows the scatter plots comparing normalization factors (NFs) from single genes (UBC, GAPDH, ACTB) or the geometric average of the expression of the three (classic) vs. the new NF based on the genes identified by this study (RPL13, RPL31, RPL37, RPL30). The lower panel shows the estimate of the Pearson correlation r (the black dot) and its 95% confidence interval (vertical black line) for each of the scatter plots above. All the confidence intervals include zero (horizontal black line), therefore we can conclude there is not enough evidence of a correlation between any of the classical NFs and the new NF. **(B)** Level of expression (qPCR) for UBC as dCt using the new NF. The upper panel shows the expression in three biological replicates (three independent ECFC clones) in control vs. quiescence (matched samples). The lower panel shows the expression in four biological replicates (four independent ECFC clones) in young vs. late passage (LP) (matched samples). The horizontal red line corresponds to the estimate of the average expression in the treatment group. The vertical red line is the 95% confidence interval of the estimate of the average expression. **(C)** Level of expression (qPCR) for IGFBP7 (upper panel) and IL6 (lower panel) as dCt, using either the new NF (right side) or the classical NF (left side) in four biological replicates (matched samples). IGFBP7 is significantly upregulated (as expected comparing LP vs young cells) using both the classical NF and the new NF. IL6 is significantly upregulated (as expected comparing LP vs young cells) only using the new NF. The horizontal red line corresponds to the estimate of the average expression in the treatment group. The vertical red line is the 95% confidence interval of the estimate of the average expression. *p < 0.05; ns, not significant.

Although we followed stringent criteria to select optimal housekeeping genes for ECFC senescence studies, it is difficult to define the “real normalizer”; therefore, we decided to compare our set of candidate housekeeping genes with a set of “classical” housekeeping genes including ACTB, UBC, and GAPDH in terms of their normalization factor. The objective was to test if our set of housekeeping genes provides a normalization factor which is not correlated with the one obtained by using the classical housekeeping genes (therefore different). Correlation analysis demonstrated that the normalization factor using our set of selected housekeeping genes is not significantly correlated with any normalization factor provided by other classical approaches (ACTB alone, GAPDH alone, UBC alone and ACTB GAPDH and UBC altogether) (Figure 4A). Consequently, considering that our set of genes has been proven to be more stable compared to the classical ones, we can conclude that our normalization factor is more reliable than the one provided by classical housekeeping genes alone and combined. This accuracy is further underscored by different results found within our experimental setup using ECFC and senescence/quiescence *in vitro* models. The expression of UBC was significantly downregulated in quiescent ECFCs when using our housekeeping gene panel (Figure 4B). Similarly, we found significant upregulation of UBC gene expression in senescent ECFCs, confirming UBC as an unsuitable housekeeping gene for senescence and quiescence studies in ECFCs. This is in agreement with previously published data from another group investigating cell size and gene expression; which reported similar changes in UBC expression in both A549 cells and primary human fibroblasts (23). In addition, we evaluated gene expression of IGFBP7 and IL6, which are components of the senescent associated secretory phenotype (SASP) and were expected to be upregulated. While both, our new and the classical housekeeping panels identified a significant upregulation of IGFBP7, only our new housekeeping panel was able to detect a significant upregulation of IL6 (Figure 4C). Taken together, we were able to validate our set of new housekeeping genes as highly accurate for ECFC senescence studies.

## 4. Discussion

We have shown a workflow for the identification of housekeeping genes for RT-qPCR using an unbiased approach starting from a transcriptomic dataset. Considering the growing number of -omic dataset freely available online, we demonstrate that relying on classically reported housekeeping gene is not always accurate. Moreover, with this approach, it is possible to identify candidates for specific cell types and biological processes that are not yet validated for RT-qPCR experiments. Here, we have used such analytical workflow to identify the optimal housekeeping panel for studies investigating ECFCs and cellular senescence. The use of a non-stable reference gene leads to incorrect results. As established by MIQE guidelines (9), reference gene validation must be carried out for every experiment and every type of sample, whether treated or not. In this paper, we propose a panel of the most stable genes to be used in ECFCs using different means of inducing senescence. This study has identified and validated the genes RPL13, RPL31, RPL37, and RPL30 as the most stable in ECFCs for senescence studies. Here, we report a significant decrease in expression of the classical housekeeping gene UBC under quiescence, coupled with a significant increase under senescence, when using the novel panel. This is in agreement with previous work showing that UBC expression is reduced in fibroblasts under quiescence (23) further strengthening the argument that housekeeping genes must be optimized for each experimental condition and cell type to ensure correct interpretation of results. The optimal number of housekeeping reference genes needed for analysis of ECFCs in senescence studies was established. Although MIQE guidelines published in 2009 recommend using at least two housekeeping genes, a systematic review found that the average number of housekeeping genes used across 130 studies between 2010 and 2015 was 1.2 (13). This highlights that while MIQE guidelines are widely accepted, there is a major lack of compliance. In terms of recommendations for future studies, we feel that the use of at least three housekeeping controls is a relatively modest request to ensure that RT-qPCR data investigating senescence mechanisms in ECFCs is accurately analyzed. The mechanistic and organelle-based locations of candidate housekeeping genes was characterized in detail. We found that the majority of identified stable genes were located within the KEGG category genetic information processing, which include also genes coding for ribosomal proteins. This information highlights why a similar workflow as the outlined in this report, should be used to identify future candidates when experimental parameters or cell types change. For example, our housekeeping panel, although validated for senescence studies, will not be ideal for investigations into pathologies focussing on ribosomal proteins or genes. We want to emphasize that there is no perfect “universal” housekeeping gene control for every condition; for any experimental setup, it is essential to first identify the most effective controls. There is little available information in the literature about housekeeping genes for ECFCs; however, a recent publication identified a selection of optimal housekeeping genes for differentiating ECFCs from umbilical cord blood mononuclear cells on scaffold delivery systems (24). This investigation yielded a panel of genes different to ours (YWHAZ, GAPDH, UBC) although the experimental conditions did not investigate senescence and only utilized seven common candidate genes. To our knowledge, this is the first investigation which has used microarray data as a starting point for identifying reference gene candidates in ECFCs. Microarrays have been used before for identifying a shortlist of novel candidate genes in grapevine exposed to water and heat stress (25). In this study, a method was established for cleaning raw microarray data by excluding genes below the threshold of detection for qPCR and those genes which would be unsuitable due to high variation (measured by observing the standard deviation and excluding those of which the standard deviation was unacceptable). This improves the screening process in two ways: Firstly, it allows the identification of robust candidates by excluding those which are unsuitable. Secondly, it reduces the computational burden required to run the algorithm for identifying genes. This is a particular disadvantage of geNorm, where the algorithm follows a polynomial time kinetic (Figure S2). This study will contribute to the current field by providing an in-depth methodology for identifying potential housekeeping candidates for ECFCs from microarray data. Although the authors chose senescence as the focus for the analytical pipeline, the methodology is transferable to other cell types, pathologies, and experimental settings. Other cell types, such as human umblical vein endothelial cells (HUVECs) and human mesenchymal stem cells (hMSCs), have already undergone rigorous housekeeping gene selection from a broad range of sources (26, 27) including microarrays. As such, it is important that ECFCs are held to the same rigorous standards. As ECFCs are a promising candidate for cellular therapy applications, it is paramount to determine the mechanisms which may disrupt their function in pathological conditions. For example, it is important to study ECFCs in the context of hypoxia, high glucose, inflammation, oxidative stress, among others. Since senescence of endothelial progenitors is a major component driving vascular aging, this study decided to focus on housekeeping genes for ECFCs when evaluating their senescence programme. Results shown here have practical applications for future studies investigating ECFC senescence. This study also recommends screening transcriptomic changes as a valid strategy to identify precise reference controls in any future ECFC studies.

## Author Contributions

KM and EP contributed equally to this manuscript. KM and EP carried out the experiments. JG-F generated samples for the microarray. RM, KM, and EP wrote the manuscript with support from JG-F. MM provided technical advice. RM conceived the original idea.

### Conflict of Interest Statement

The authors declare that the research was conducted in the absence of any commercial or financial relationships that could be construed as a potential conflict of interest.
