# Supplementary material for: Selection of a Real-Time PCR Housekeeping Gene Panel in Human Endothelial Colony Forming Cells for Cellular Senescence Studies
Source: Front Med (Lausanne). 2019 Mar 11;6:33. doi: 10.3389/fmed.2019.00033 (PMC6421261; doi:10.3389/fmed.2019.00033)
Supplement: Supplementary file 1 [file Table_1.pdf]

Ensembl	GeneSymbol	FW	REV
ENSG00000145592	RPL37	CAAGCGCAAGAGAAAGTATAACTGG	CAGCTGCCCTCTTGGGTTTAG
ENSG00000213741	RPS29	AGGGTTCTCGCTCTTGTCGT	TGCCCCGGATAATCCTCTGAA
ENSG00000163682	RPL9	TGCGTCTACTGCGAGAATGAAG	CCACCATTTGTCAACCCGGA
ENSG00000026025	VIM	GCGAGGAGAGCAGGATTTCTC	AGTGGGTATCAACCAGAGGGAG
ENSG00000119013	NDUFB3	GCTGGCTGCAAAAGGGCTA	CTCCTACAGCTACCACAAATGC
ENSG00000169020	ATP5I	CAGGTCTCTCCGCTCATCAAG	GCCCGAGGTTTTAGGTAATTGT
ENSG00000071082	RPL31	CTCGGGCACTCAAAGAGATTC	CGGATTCGGTATGGCACATTC
ENSG00000177954	RPS27	ATGCCTCTCGCAAAGGATCTC	TGAAGTAGGAATTGGGGCTCT
ENSG00000118523	CTGF	ACCGACTGGAAGACACGTTTG	CCAGGTCAGCTTCGCAAGG
ENSG00000065518	NDUFB4	ATGTCGTTCCCAAAGTATAAGCC	GAAGCAGGTACTCTCGTTTCAG
ENSG00000154723	ATP5J	GTTCTCCTCTGTCATTCGGTCA	TCCAGATGTCTGTCGCTTAGAT
ENSG00000137154	RPS6	TGGACGATGAACGCAAACTTC	TTCGGACCACATAACCCTTCC
ENSG00000075624	ACTB	CATGTACGTTGCTATCCAGGC	CTCCTTAATGTCACGCACGAT
ENSG00000116459	ATP5F1	AGGTCCAGGGGTATTGCAG	TCCTCAGGGATCAGTCCATAAC
ENSG00000166441	RPL27A	TGAGGAAGACCCGGAAACTTA	GCCTGGGTGGTATTTGTCGAA
ENSG00000226784	PGAM4	TTAGCCATTTTGTCGCCTGC	TCCTCATTGGTCGTGGCTT
ENSG00000124614	RPS10	CTGCGAGACTCACAAGAGGG	CACGTCCACGACCAAATCC
ENSG00000156482	RPL30	GCTGGAGTCGATCAACTCTAGG	CCAATTTCGCTTTGCCTTGTC
ENSG00000109971	HSPA8	ACCTACTCTTGTGTGGGTGTT	GACATAGCTTGGAGTGGTTCG
ENSG00000167526	RPL13	TCAAAGCCTTCGCTAGTCTCC	GGCTCTTTTTGCCCGTATGC
ENSG00000108298	RPL19	AAAACAAGCGGATTCTCATGGA	TGCGTGCTTCCTTGGTCTTAG
ENSG00000166136	NDUFB8	CCGCCAAGAAGTATAATATGCGT	TATCCACACGGTTCCTGTTGT
ENSG00000167283	ATP5L	ATGGCCCAATTTGTCCGTAAC	TGGCGTAGTACCAAAATGTGG
ENSG00000150991	UBC	GTGGCACAGCTAGTTCCGTC	ACGAAGATCTGCATTGTCAAGT
ENSG00000170027	YWHAG	AGCCACTGTCGAATGAGGAAC	CTGCTCAATGCTACTGATGACC
ENSG00000117450	PRDX1	TCAAGCCTGATGTCCAAAAGAG	ACCGCAGCCTGGCACTAA
ENSG00000111640	GAPDH	GAGAGAGACCCTCACTGCTG	TGGTACATGACAAGGTGCGG
ENSG00000166710	B2M	AAGTGGGATCGAGACATGTAAG	GGAATTCATCCAATCCAAATGCG

Table S1 Table of primer sequences for RT-qPCR.
