# Supplementary material for: Selection of a Real-Time PCR Housekeeping Gene Panel in Human Endothelial Colony Forming Cells for Cellular Senescence Studies
Source: Front Med (Lausanne). 2019 Mar 11;6:33. doi: 10.3389/fmed.2019.00033 (PMC6421261; doi:10.3389/fmed.2019.00033)
Supplement: Supplementary file 3 [file Data_Sheet_1.pdf]

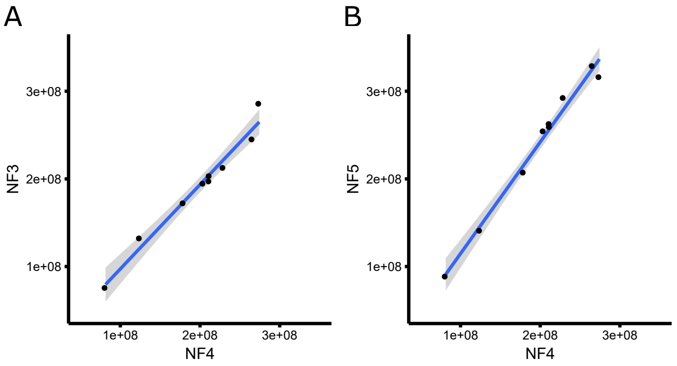

Figure S1. Selection of HK genes.(**A**) Scatter plot of new NF using 3 (y axis) vs. 4 genes (x axis). (**B**) Scatter plot of new NF using 5 (y axis) vs. 4 genes (x axis).

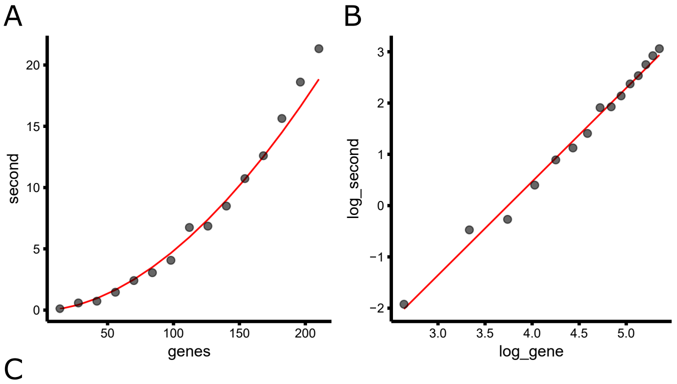

C

Architecture: x86_64
CPU op-mode(s): 32-bit, 64-bit
Byte Order: Little Endian
CPU(s): 4
On-line CPU(s) list: 0-3
Thread(s) per core: 2
Core(s) per socket: 2
Socket(s): 1
Model name: Intel(R) Core(TM) i7-5500U CPU @ 2.40GHz
Stepping: 4
CPU max MHz: 3000.0000
CPU min MHz: 500.0000
L1d cache: 32K
L1i cache: 32K
L2 cache: 256K
L3 cache: 4096K

Figure S2. Benchmark geNorm algorithm.(**A**) Kinetics for time complexity (time required to run an algorithm) of geNorm algorithm. Graph shows number of genes versus time. (**B**) Same data used in **A** plotted in log scales. The plot confirms geNorm as a polynomial time algorithm. (**C**) Specs of the machine used for the benchmark.
